# Value of serum and induced sputum surfactant protein-D in chronic obstructive pulmonary disease

**DOI:** 10.1186/2049-6958-8-36

**Published:** 2013-06-01

**Authors:** Berna Akinci Ozyurek, Sevinc Sarinc Ulasli, Serife Savas Bozbas, Nilufer Bayraktar, Sule Akcay

**Affiliations:** 1 Ataturk Chest Diseases and Thoracic Surgery Training and Research Hospital, Ankara, Turkey; 2 Department of Pulmonary Diseases, Afyon Kocatepe University Faculty of Medicine, Afyon, Turkey; 3 Faculty of Medicine, Department of Pulmonary Diseases, Baskent University, Ankara, Turkey; 4 Faculty of Medicine, Department of Biochemistry, Baskent University, Ankara, Turkey

**Keywords:** Chronic obstructive pulmonary disease, Induced sputum, Surfactant protein-D

## Abstract

**Background:**

Surfactant Protein D (SP-D) is an important marker in chronic obstructive pulmonary disease (COPD). Serum SP-D levels increase while lung production of SP-D decreases in COPD. SP-D is a specific biomarker for monitoring COPD, assessment of exacerbation frequency and arrangement of treatment modalities. In the present study, we aimed to investigate the correlation between serum and induced sputum SP-D levels with severity and acute exacerbations of COPD.

**Method:**

20 healthy subjects, older than 40 years, with at least 10 pack/years smoking history (group 1), 20 stage I-II COPD patients (group 2) , and 20 stage III-IV COPD patients (group 3) were enrolled in the study. All subjects performed pulmonary function tests. Venous blood samples were taken to determine complete blood count, C-reactive protein(CRP) and serum SP-D levels. Induced sputum samples were obtained to determine SP-D level. COPD patients were followed up for acute exacerbations for 6 months.

**Results:**

Serum SP-D levels of group 3 were the highest and induced sputum SP-D levels of group 2 were the lowest among the three groups. SP-D levels of induced sputum decreased in patients with increasing number of cigarette pack/years (p = 0.03, r = −0.115), whereas serum SP-D levels increased in these patients (p = 0.0001, r = 0.6 ). Induced sputum SP-D levels in COPD patients receiving inhaled corticosteroid treatment were significantly higher than in patients who were not receiving inhaler corticosteroid treatment (p = 0.005). An inverse correlation between serum SP-D levels and FEV_1_ (%) was found and there was a positive correlation between the serum SP-D levels and exacerbations frequency in 6-month follow up period (p = 0.049 ,r = −0.252; p = 0.0001, r = 0.598 respectively).

**Conclusion:**

Our study demonstrates the adverse effects of smoking on local SP-D levels since low levels of induced sputum SP-D were found in the group of current smokers, who were not receiving inhaled corticosteroid treatment. Relationship between serum SP-D and COPD exacerbations frequency suggests that serum SP-D level may be used as a lung-specific biomarker during the follow up and progression of COPD.

## Background

Chronic obstructive pulmonary disease (COPD) is a complex chronic inflammatory disease that involves the activity of various inflammatory cells and mediators [1]. Both local and systemic inflammatory reactions are observed in COPD. There are ongoing researches trying to find out different markers in COPD course. For instance, surfactant protein D (SP-D) is one of the most frequently studied markers contributing to immune and inflammatory regulation within lungs. SP-D is a 43kDa member of the collectin family that is produced from collagenous glycoprotein in type II pneumocytecells. SP-D is a protein responsible for homeostasis which has an important protective role in the immune system against inhaled microorganisms and allergens. It plays a part in protection against viral, bacterial, and fungal infections, as well as apoptotic cells [2]. Serum SP-D concentration exhibits an increase together with the decrease in bronchoalveolar lavage in COPD. Furthermore, serum SP-D level and FEV_1_ display a negative correlation in COPD [3]. Higher serum SP-D levels have been found [3] in advanced COPD cases with worsening health and aggravating shortness of breath. SP-D is thought to play a role in the pathogenesis and progression of COPD [4]. SP-D level declines as the disease progresses. There are also some studies indicating the increase of BAL SP-D level using inhaled corticosteroids. The association between decreased SP-D level in lungs and smoking has also been demonstrated in previous studies [5,6]. Therefore, SP-D is a promising biomarker that might help to determine the health status of patients with lung diseases, particularly with respect to progression of dyspnea and decreasing pulmonary functions.

SP-D of serum and BAL or induced sputum may be used as a lung specific biomarker in the assessment of COPD progression and management. The number of studies investigating the relationship between local and systemic SP-D levels with COPD is not as high as it should be. Therefore, in the present study we aim to investigate the relationships between COPD severity and acute exacerbations frequency with serum and induced sputum SP-D levels.

## Methods

This study was approved by the Research Committee of the Medical Faculty and Health Sciences, Baskent University (project# KA09/277) and supported by the Research Fund of Baskent University. Written informed consent was obtained from each participating patient and control subjects prior to the study.

Our study sample comprised of 60 subjects in total: a control group including 20 individuals (16 smokers and 4 ex-smokers) above 40 years of age (mean age: 44.85 ± 5.80 years) and with a history of at least 10 pack year cigarette consumption (mean cigarette pack year: 23 ± 13 pack year) (Group 1), 20 mild and moderate COPD patients (Group 2), and 20 severe and very severe COPD patients (Group 3) based on GOLD classification [7].

The patients below 40 years of age and those with a history of COPD exacerbation/infection within the last 4 weeks, cardiac disease, chronic liver and kidney failure, asthma, malignancy, hypertension, diabetes mellitus, and any additional medical disorders were excluded from the study.

In our study all patients and control subjects performed pulmonary function tests (PFT). Furthermore, venous blood sample was collected from each participant for evaluating complete blood count (CBC), C-reactive protein (CRP), and serum SP-D level. Induced sputum specimen was obtained and also tested for induced sputum SP-D level. COPD patients were followed for 6 months to determine exacerbation frequency. Acute exacerbation of COPD was defined as a sustained (lasting 48 hours or more) worsening of dyspnea, cough or sputum production leading to an increase in the use of maintenance medications and/or supplementation with additional medications [7,8]. The data concerning the exacerbation frequency were collected via hospital visits and telephone calls.

### Measurement of biochemical parameters

Venous blood sample was obtained from each patient. 3 ml venous peripheral blood specimen was put into a tube with K_3_-EDTA for CBC and 5 ml venous peripheral blood specimen was put into an additive-free tube for analysis of CRP and SP-D levels. Serum samples were prepared by collecting blood in a vacuum tube and allowing it to clot for 30 minutes at room temperature. About 1mL of serum was obtained after centrifugation at 1100g for 10 minutes and stored in small aliquots at −80°C until analysis. SP-D level was studied by ELISA immunoassay method. Serum CRP level was measured by an ultrasensitive latex-enhanced immunoassay method, using CRP Ultra reagent (Sentinel Diagnostics, Milan, Italy) in Abbott Architect C8000 Analyzer according to the manufacturer’s specifications. The detection limit was 0.2 mg/L. The inter- and intra-assay variability were 8.22% and 4.84%, respectively. CBC was carried out with Abbott Cell-Dyne® 3700 System device (Abbott Diagnostics, Santa Clara, CA, USA).

### Pulmonary function test

Pulmonary function test was performed with a clinical spirometer (SensorMedicsVmax spectra 229, Bilthoven, The Netherlands) according to the ERS standards [9]. We carried out forced vital capacity (FVC) and forced expiratory volume (FEV_1_) measurements, and calculated the FEV_1_/FVC ratio. Total lung capacity (TLC), residual volume (RV), functional residual capacity (FRC), and vital capacity (VC) were measured by the multiple nitrogen washout method. Twenty healthy individuals having normal PFTs and 40 patients diagnosed as mild, moderate, severe, and very severe COPD based on the current Global Initiative for Chronic Obstructive Lung Disease (GOLD) guidelines using postbroncodilatator FEV_1_ values (post bronchodilator values were obtained after 400 mcg salbutamol inhalation), were enrolled in the study [7].

### Induced sputum analysis

The patients were informed about the process and FEV_1_ values were measured before the bronchodilatation. Then, 10 minutes after inhaling 400 μg salbutamol, post-bronchodilatator FEV_1_ values were measured. Induction was started with 3% NaCl in patients with FEV_1_ value >1 L or >60%. This was continued for a period up to 20 minutes by incremental doses applied with nebulizer at 5 minute intervals and FEV1 values were measured after each induction. If 20% or more decrease in FEV1 values was detected, process was terminated.

The induction started using 0.9% NaCL with nebulizer in patients with a FEV_1_ value <1 L or <60%. The induction was carried out for 30 seconds, 1 minute, and 5 minutes. At the end of these time intervals, FEV_1_ value was re-measured and induction was discontinued if there was a decrease of 20% or more. Induction with higher concentration was not applied to patients when adequate material could be obtained at this concentration. If the patient failed to produce sputum and/or adequate sputum at these concentrations, induction was applied using 3% NaCl with nebulizer and it was carried out for 30 seconds, 1 minute, and 2 minutes. At the end of these time intervals, FEV_1_ value was measured again and induction was discontinued if there was a decrease of 20% or more. Induction with a higher concentration was not applied to patients when adequate material could be obtained at this concentration. If the patient failed to produce sputum and/or adequate sputum at this concentration, induction was applied with 4.5% NaCl and it was carried out for 30 seconds, 1 minute, 2 minutes, and 4 minutes. At the end of these times, FEV_1_ value was measured again and induction was discontinued if there was a decrease of 20% or more. Following each induction, participants were asked to rinse their mouth with water and produce sputum by cough.

### The processing of induced sputum

The induced sputum was immediately analyzed following the acquirement. Firstly, the specimen was weighed. Sputum was treated with Sputalysin (0.1% DTT) by mixing the agent in a 1:1 ratio with the specimen. The mixture was vortexed for 15 minutes at room temperature and thereafter it was filtered through a 48μm-thick nylon filter. The resulting filtrate was weighed. The cell viability of the filtrate was evaluated by Trypan blue and total cell count (TCC) was calculated on a Thoma slide. TCC was calculated by following the formula: Average number of cells in one large square x dilution factor* x 10^4^ (*dilution factor is 2x2 = 4 (1:1 dilution with Sputalysin and 1:1 dilution with trypan blue)). The filtrate (790μg) was centrifuged at 4°C for 10 minutes. The resultant supernatant was separated and put into Falcon tubes. These tubes were stored at −20°C.

### Induced sputum and serum SP-D level determination

Surfactant protein D concentration in serum and induced sputum samples was determined by a sandwich enzyme-linked immunosorbent assay (ELISA) system (SP-D; Biovendor, Brno, Czech Republic). For SP-D, the inter- and intraassay CV were 3.7% and 2.3%, respectively, and the sensitivity was 0.01 ng/ml.

### Statistical analysis

The statistical analyses of our study were performed using SPSS statistical software version 15.0. The variables were investigated using visual (histograms, probability plots) and analytical methods (Kolmogorov-Simirnov test) to determine the normality of distributions. The results were expressed as mean ± standard deviation and median value. For continuous variables without normal distribution Mann–Whitney U test was used for the comparison of the two groups (patients receiving inhaler steroids or not receiving inhaler steroids), whereas Kruskal-Wallis test for the comparison of parameters between 3 groups. T- test was used for the comparison of parameters with normal distribution between 2 groups and ANOVA together with Bonferroni correction was used for the comparison of continuous parameters with normal distribution among the three groups. The parameters affecting 6 month exacerbation frequency, induced sputum and serum surfactant protein D levels were investigated using Spearman correlation analysis. Multiple linear regression models were used to identify independent predictors of 6 month exacerbation frequency, serum and induced sputum surfactant protein D levels. The model fit was assessed using appropriate residual and goodness of fit statistics. A 5% type-I error level was used to infer statistical significance.

## Results

The demographic characteristics, PFT parameters and complete blood count results of our study groups are reported in Table 1. CRP levels of group I were the lowest among study groups (p = 0.03).

**Table 1:** Demographic characteristics and respiratory function test results of the study population

	**Group 1 (control subjects)**	**Group 2 (stage 1 and 2 COPD patients)**	**Group 3 (stage 3 and 4 COPD patients)**	**P**
	**(n = 20)**	**(n = 20)**	**(n = 20**)	
**Mean Age (year)**	44.85 ± 5.80	60.35 ± 9.40	63.70 ± 8.60	**p = 0.001**
**Gender (F/M)**	8/12	0/20	3/17	**p = 0.004**
**Mean BMI (kg/m**^**2**^**)**	33.36 ± 5.70	32.8 ± 6.70	29.6 ± 5.90	p = 0.148
**Smoking status**				
**Active smokers**	16 (80%)	10 (50%)	2 (10%)	**p = 0.001**
**Ex-smokers**	4 (20%)	10 (50%)	18 (90%)	
**Cigarette pack years**	23 ± 13 (20)	51 ± 28 (45)	51 ± 33(45)	**p = 0.001**
**Inhaled steroid use**	0	6	18	**p = 0.001**
**Mean FVC (%)**	131.5 ± 15.1	101.7 ± 19	76.7 ± 16.8	**p = 0.001**
**Mean FEV**_**1 **_**(%)**	123 ± 14	75.7 ± 16.4	39.7 ± 7.24	**p = 0.001**
**Mean FEV**_**1**_**/FVC (%)**	78.9 ± 4.8	59.75 ± 10.8	43.3 ± 11.3	**p = 0.001**
**Mean FEF**_**25-75**_**(%)**	94.5 ± 23.4	33.5 ± 18.6	12.5 ± 4.54	**p = 0.001**
**Mean VC (%)**	130 ± 16.9	94.5 ± 19	80.8 ± 19	**p = 0.001**
**Mean RV (%)**	120 ± 29	156 ± 37	150 ± 77	**p = 0.001**
**Mean SpO2 (%)**	96.6 ± 1.1	94 ± 1.9	92.1 ± 2.1	**p = 0.001**
**Mean CRP level (mg/L)**	2.52 ± 2.4	8.3 ± 10	8.1 ± 9.49	**p = 0.014**
**Mean Hb (g/dl)**	14.21 ± 1.55	14.7 ± 1.43	14.6 ± 14	p = 0.282
**Mean WBC (/μL)**	7.449 ± 1.704	7.291 ± 1.084	8.402 ± 1.694	p = 0.102
**Mean PMNL (/μL)**	4.272 ± 1.166	4.310 ± 956	5.519 ± 1.205	**p = 0.003**

The results are expressed as mean ± standard deviation.

*BMI* Body mass index, *CRP* C-reactive protein, *FEV*_*1*_ forced expiratory volume, *FRC* functional residual capacity, *FVC* forced vital capacity, *Hb* hemoglobin, *PMNL* polymorphonucleer leukocyte, *RV* residual volume, *TLC* total lung capacity, *VC* vital capacity, *WBC* white blood cell count.

Smoking status (active, ex-smoker) of the groups was also evaluated. 80% of the healthy individuals (n = 16) in Group 1, 50% of patients (n = 10) in Group 2, and 10% of patients (n = 2) in Group 3 were active smokers. The amount of cigarette consumption (pack/years) was 20 pack/years in Group 1, 45 pack/years in Group 2, and 45 pack/years in Group 3. When we asked the ongoing bronchodilatator and/or oxygen therapy of COPD patients, 24 patients were found to be on inhaled corticosteroid therapy and 12 patients on nasal oxygen therapy due to type I respiratory failure (Table 2). Mean duration of inhaled steroid treatment was 5.31 ± 3.81 years.

**Table 2:** Treatment modalities of COPD patients

	**Group 2 (stage 1 and 2 COPD patients)**	**Group 3 (stage 3 and 4 COPD patients)**
	**(n = 20)**	**(n = 20)**
Short acting β_2_ agonist	6	12
Long acting β_2_ agonist	12	18
Long acting anticholinergic	7	15
Inhaler steroid	6	18
N-acetyl systein	2	7
Long term oxygen therapy	2	10
Pulmoner rehabilitation	0	7

The serum and sputum SP-D values of subjects in all three groups were compared (Table 3). Serum SP-D levels of Group 3 were the highest and serum SP-D levels of Group 2 were higher than Group 1. However, there was no statistically significant difference among the three groups in terms of serum SP-D level (p = 0.099). In addition, we did not find statistically significant difference among the three groups in terms of induced sputum SP-D levels (p = 0.836). Although, induced sputum SP-D levels in Group 2 were the lowest, they did not achieve to the significance level.

**Table 3:** Comparison of SP-D levels and cell viability among the study groups

	**Group 1 (control subjects)**	**Group 2 (stage 1 and 2 COPD patients)**	**Group 3 (stage 3 and 4 COPD patients)**	**p**
	**(n = 20)**	**(n = 20)**	**(n = 20)**	
Serum SP-D (ng/ml)	86.2 ± 49	108.9 ± 65	129 ± 71	p = 0.10
Sputum SP-D (ng/ml)	33.1 ± 34	28 ± 15.2	30.42 ± 12.8	p = 0.83
Viability %	73.5 ± 12.1	79.8 ± 11.2	80.1 ± 19	p = 0.86

The results were expressed as mean ± standard deviation.

*COPD* Chronic obstructive pulmonary disease, *SP-D* surfactant protein D.

The groups were also evaluated in terms of relationships between cigarette consumption (pack year) and serum/sputum SP-D levels. While sputum SP-D and cigarette consumption demonstrated a negative correlation (p = 0.03, r = −0.115), serum SP-D and cigarette consumption showed a positive correlation (p = 0.0001, r = 0.6 ).No significant difference was observed between genders in terms of serum/sputum SP-D levels and cell viability.

Serum SP-D levels were significantly correlated with FEV1 (%) (p = 0.049; r = −0.252). However no significant correlation was found between FEV1 (%) and induced sputum SP-D levels (p = 0.92, r = −0.013).

Induced sputum and serum SP-D levels were significantly different between patients with and without inhaled corticosteroid therapy (p = 0.005 and 0.038 respectively). The COPD patients receiving inhaled corticosteroid therapy had higher SP-D levels compared to those not receiving inhaled corticosteroid therapy (Table 4). The Number of years on inhaled steroid treatment was significantly correlated with induced sputum SP-D levels whereas no significant correlation was evidenced with serum SP-D levels (p = 0.0001, r = 0.800; p = 0.59, r = 0.104 respectively).

**Table 4:** Comparison of patients with and without inhaled corticosteroid therapy

	**COPD patients receiving inhaled steroid (n = 24)**	**COPD patients not receiving inhaled steroid (n = 16)**	**p**
Serum SP-D (ng/ml)	132 ± 71	99 ± 59	**0.038**
Sputum SP-D (ng/ml)	35.5 ± 14	20 ± 7.8	**0.005**

The results were expressed as mean ± standard deviation.

*COPD* Chronic obstructive pulmonary disease, *SP-D* surfactant protein D.

We also evaluated the relationships between serum/sputum SP-D levels and 6-month exacerbation frequency in COPD patients of Groups 2 and 3. There was no significant correlation between sputum SP-D levels and 6-month exacerbation frequency (p = 0.051; r = 0.342). However, patients with increased serum SP-D levels had significantly higher 6-month exacerbation frequency (p = 0.0001; r = 0.59). The other parameters such as BMI, cigarette pack years, FEV1(%) and number of years on inhaled steroid treatment were not correlated with 6-month exacerbation frequency (p = 0.39, r = 0.152; p = 0.117 r = −0.278; p = 0.177, r = −0.241; p = 0.137, r = 0.294 respectively).

Multiple linear regression analyses were conducted after adjusting for age, cigarette pack years, BMI, FEV1 (L), FEV1(%), number of years on inhaled steroid treatment, serum and induced sputum SP-D levels; only serum SP-D levels were found to be significantly associated with acute exacerbation frequency (p =0.0001). Sputum surfactant protein D level was found to be significantly associated with number of years on inhaled steroid treatment (p = 0.0001). Serum surfactant protein D level was significantly related with 6 month exacerbation frequency (p = 0.0001) (Table 5).

**Table 5:** Multiple linear regression models


Multiple linear regression model for six month exacerbation frequency by serum and induced sputum SP-D levels				
	Coefficient B	Standard error	t	p
Constant	−0.37	0.32	−1.15	0.25
Serum SP-D levels	0.008	0.002	4.22	**0.0001**
Sputum SP-D levels	0.011	0.009	1.18	0.247
Multiple linear regression model for induced sputum SP-D levels by six month exacerbation frequency and number of years on inhaled steroid treatment.				
	Coefficient B	Standard Error	t	p
Constant	16.948	3.78	4.4	0.0001
Six month exacerbation frequency	1.38	2.14	0.64	0.525
Number of years on inhaled steroid treatment	2.848	0.53	5.37	**0.0001**
Multiple linear regression model for serum SP-D levels by age, FEV1 (%) and six month exacerbation frequency				
	Coefficient B	Standard error	t	p
Constant	17.660	70.64	0.25	0.80
Age	1.461	1.08	1.35	0.187
FEV1(%)	−0.61	0.45	−1.36	0.182
Six month exacerbation frequency	42.598	9.85	4.32	**0.0001**

*FEV*_*1*_ forced expiratory volume, *SP-D* surfactant protein D.

## Discussion

In this study, we investigated the relationships between local and systemic SP-D levels, and course of COPD by evaluating serum and induced sputum SP-D levels. In the literature review, no study including both induced sputum and serum SP-D measurement in COPD patients has been found according to our knowledge.

Induced sputum analysis has been recently recognized as a valuable method for revealing the pathogenesis of inflammatory airway diseases such as COPD and asthma, and monitoring the activity and treatment response in these pathologies [10-13]. Currently, induced sputum is recognized to reflect lower respiratory tract inflammation [10,13]. Therefore, we preferred to use induced sputum specimens, also known as cost-effective diagnostic tool, in evaluating the courses of local and systemic inflammation in COPD.

Previous studies have demonstrated a positive correlation between cigarette consumption (pack year) and serum SP-D levels. Serum SP-D concentrations have been found to be higher in current smokers than in ex-smokers [2,14]. In our study, among COPD patients, there was a negative correlation between induced sputum SP-D levels and cigarette consumption (pack year), whereas serum SP-D levels and cigarette consumption (pack year) showed a positive correlation.

The comparison of induced sputum SP-D levels among three groups revealed no statistically significant difference. Sputum SP-D concentrations of Group 2 were lowest among study groups. Furthermore, 50% of patients in Group 2 were currently active smokers. Inhaled corticosteroid treatment was more common in Group 3 due to increased airway obstruction level and exacerbation frequency of severe and very severe COPD patients. Sputum SP-D levels were significantly different between patients with and without inhaled corticosteroid therapy in the present study. Moreover, significant correlation was also found between number of years on inhaled corticosteroid treatment and induced sputum SP-D levels in linear regression analysis. These results also support the fact that the use of inhaled corticosteroids indirectly contributes to the local SP-D levels in a positive way [15]. Ishikawa et al. found increased induced sputum SP-D levels in 28 COPD patients compared to subjects with prolonged cough [16]. They included patients with mild, moderate and severe COPD patients, and subjects with prolonged cough as control group and did not mention the medications of the subjects. Different results might be due to the fact that study populations and medications in the study by Ishiwaka et al. were different from those in present study.

In COPD patients, serum SP-D levels and FEV_1_ show a negative correlation, whereas lung SP-D levels and FEV_1_ exhibit a slightly positive correlation [3]. In the present study, serum SP-D levels were negatively correlated with FEV_1_(%) and group 3 had the highest serum SP-D levels among study groups. Group 2 had higher serum SP-D levels than Group 1, although, these differences did not reach to significance level. We believe that low number of patients in all groups was a limitation for the statistical comparison.

We had a higher number of males than females in study groups (F/M:11/49). In a recent study COPD rate was reported as four times higher in males than females in our country and total smoking, biomass, and occupational exposure were also found to be overwhelmingly higher in males than females (16.1% and 3.9% respectively) [17]. Therefore, these results could be attributed to a higher incidence of COPD in males as higher incidence of smoking and environmental/occupational exposure in our country.

Acute exacerbations that are observed during the course of the COPD are significant causes of morbidity and mortality [18,19]. Some COPD patients exhibit a higher acute exacerbation frequency mostly due to the infections of tracheobronchial tree. Some studies demonstrate that increased airway inflammation incidence may have a role in the elevated acute exacerbation frequency [18]. Our patients were followed up for 6 months for acute exacerbations. In 20 (50%) of our COPD patients in group 2 or 3, the acute exacerbation frequency varied between 1 and 3 within a 6 month follow-up period. 85% of 20 patients with a history of exacerbation were moderate, severe, and very severe COPD cases. This finding is consistent with the fact that raised airway inflammation increases the frequency and number of acute exacerbations.

In advanced COPD patients especially with frequent acute exacerbations, markers of systemic inflammation such as cytokines, chemokines, and acute phase proteins have been used [18-20]. SP-D, has also been proposed to be a lung-specific biomarker in COPD cases [21]. Elevated serum SP-D levels can show the poor health status of COPD patients within a 3 month period [3]. Also Shakoori TA et al. demonstrated in COPD patients higher levels of serum SP-D levels during acute exacerbation than during stable period [22]. In our study, we determined a significant relationship between serum SP-D level and number of acute exacerbations within a 6 month period. In addition, we strengthen our hypothesis in linear regression analysis and found that the only significant determinant of 6 month exacerbation frequency was serum SP-D. These findings also suggest that SP-D level may be a lung-specific biomarker that can be used for monitoring COPD and its progression.

### Limitations

The limitations of our study were the unequal number of female and male patients, the absence of a non-smoker healthy control group, and the failure to convince higher number of patients to participate in the study. Further studies with larger sample sizes are needed to confirm and explore the findings of the present study.

## Conclusions

In conclusion, we believe that our preliminary study demonstrates a significant relationship between serum SP-D and COPD exacerbation frequency which suggests that serum SP-D level may be used as a lung-specific biomarker during the follow-up and progression of COPD.

## Competing interests

The authors declared that they have no competing interests.
